# The N1-suppression effect for self-initiated sounds is independent of attention

**DOI:** 10.1186/1471-2202-14-2

**Published:** 2013-01-03

**Authors:** Jana Timm, Iria SanMiguel, Katja Saupe, Erich Schröger

**Affiliations:** 1 Institute of Psychology, University of Leipzig, Seeburgstr. 14-20, Leipzig, D-04103, Germany

**Keywords:** Event-related brain potentials, Sensory suppression, Auditory N1 component, Attention, Predictive processing

## Abstract

**Background:**

If we initiate a sound by our own motor behavior, the N1 component of the auditory event-related brain potential (ERP) that the sound elicits is attenuated compared to the N1 elicited by the same sound when it is initiated externally. It has been suggested that this N1 suppression results from an internal predictive mechanism that is in the service of discriminating the sensory consequences of one’s own actions from other sensory input. As the N1-suppression effect is becoming a popular approach to investigate predictive processing in cognitive and social neuroscience, it is important to exclude an alternative interpretation not related to prediction. According to the attentional account, the N1 suppression is due to a difference in the allocation of attention between self- and externally-initiated sounds. To test this hypothesis, we manipulated the allocation of attention to the sounds in different blocks: Attention was directed either to the sounds, to the own motor acts or to visual stimuli. If attention causes the N1-suppression effect, then manipulating attention should affect the effect for self-initiated sounds.

**Results:**

We found N1 suppression in all conditions. The N1 per se was affected by attention, but there was no interaction between attention and self-initiation effects. This implies that self-initiation N1 effects are not caused by attention.

**Conclusions:**

The present results support the assumption that the N1-suppression effect for self-initiated sounds indicates the operation of an internal predictive mechanism. Furthermore, while attention had an influence on the N1a, N1b, and N1c components, the N1-suppression effect was confined to the N1b and N1c subcomponents suggesting that the major contribution to the auditory N1-suppression effect is circumscribed to late N1 components.

## Background

It is important to differentiate sensory information resulting from one’s own actions from environmental events which are not the result of our own actions. It has been proposed that this differentiation is based on an internal forward model [1-3], an idea that relates to the reafference principle [4] and the concept of corollary discharge [5] in physiological literature. Specifically, when a movement is executed, a copy of the current motor command (efference copy) is used to make predictions of the sensory consequences of the movement (corollary discharge). This sensory prediction is then compared with the actual sensory feedback. If the two correspond, sensory responses are attenuated, thereby enabling a differentiation between the sensory consequences of one’s own actions and the actions of others. Such sensory attenuation for self-generated compared to externally-generated sensations - as an index of an internal predictive mechanism - has been widely investigated in psychophysical research [6,7].

Within this self-generation framework, the N1 suppression paradigm has become a popular approach to investigate predictive auditory sensory processing [8-14]. In this paradigm, participants listen to sounds that are either initiated by their own button presses, or externally initiated. The N1 component of the event-related brain potential (ERP) is attenuated for the sounds that were self-initiated compared to the externally-initiated sounds. This N1-suppression effect has been explained as the result of an underlying predictive mechanism. In the traditional blocked version of this paradigm, self-initiated sounds and externally-initiated sounds are presented in different blocks, bearing several caveats that obscure an unambiguous interpretation in terms of the predictive coding framework [15]. For example, it seems possible that the participants’ arousal level differs between the active condition in which participants initiate the sound by their own motor behavior and the passive condition in which participants simply listen to the externally-initiated sounds. In a modified so-called mixed N1 suppression paradigm self-initiated and externally-initiated sounds are presented within the same block. Thus, sustained arousal differences between self- and externally-initiated sounds are eliminated. Studies using this paradigm also yielded (an even larger) N1-suppression effect for self-initiated sounds [16,17]. This demonstrates that the N1-suppression effect seems to occur selectively for self-initiated sounds and seems not to be caused by different arousal levels in active and passive conditions of the blocked design.

Although sustained differences in arousal are well controlled in this mixed design, it is obvious that transient arousal effects cannot be controlled for. Even more important, the improved paradigm has not been designed for excluding attentional influences on the N1-suppression effect. In fact, an enlarged P3a to externally-initiated sounds compared to the P3a for self-initiated sounds reported for the mixed design [16] suggests that externally-initiated sounds received more attention. As the N1 is known to increase with attention [18-22], it seems well possible that differences in the N1 between self- and externally-initiated sounds were in fact caused by a difference in attention directed to self- and externally-initiated sounds. The cognitive psychologist’s silver bullet to test for an attentional confound on an effect of interest (here, the N1-suppression effect) is to vary the allocation of attention over several levels and determine its influence on the effect [23,24]. Therefore, we measured the N1-suppression effect with the mixed design^a^ and manipulated the allocation of attention between blocks comprising three different attention conditions: While participants are performing the self-initiation task, attention is directed either to the sounds, the motor acts or to visual stimuli. Less attention should be directed to the sounds when participants attend to the motor act or to the visual stimuli than when they attend to the sounds. If the N1-suppression effect critically depends on an attentional difference, no (or a reduced) N1 suppression should occur when equating attention to externally and self-initiated sounds. In contrast, if N1 suppression for self-initiated sounds reflects a genuine suppression effect rather than an attentional difference, we expect comparable N1 suppression in all three attention conditions, supporting the assumption of an underlying genuine internal predictive mechanism.

Moreover, in order to focus on effects that truly reflect attenuation of sensory responses due to a match of incoming stimulation with predicted stimulation in sensory cortex, we will make a more detailed analysis of the auditory N1, separating suppression effects for the N1a, N1b, and N1c components [25,26]. It is well known that sensory and non-sensory (unspecific) components contribute to the auditory N1 [25]. Importantly, only sensory components with sources in auditory cortex are tangentially oriented, showing a fronto-central distribution with polarity inversion at the mastoids. Contrary, the unspecific component, which reflects the orienting response, appears slightly later in time than tangential components and shows no polarity reversal at the mastoids, as it does not originate in auditory cortex. If the N1-suppression effect truly reflects attenuation of sensory responses that match internal sensory predictions, then sensory-specific components generated in auditory cortex should be attenuated. If on the contrary the N1-suppression effect mostly reflects differences in the orienting response generated by self- and externally-initiated sounds then the unspecific N1 component should be most affected. Finally, by comparing the N1-suppression effects due to self-initiation and the N1-attention effects, we can determine whether the predictive modeling (putatively) underlying the N1 suppression resembles attention effects. Indeed, previous research has reported attention in time effects that share characteristics of attention to other feature effects [20,27].

## Results

### Behavioral data

Table 1 summarizes the behavioral results for the self-initiation task (inter-press time intervals, total number of button presses, timing errors) and the attention task (counting rates) obtained in the three attention conditions (*AS*, *AM*, *AV*). For the self-initiation task the analysis revealed no main effect of *Attention* for inter-press time intervals [*F*(2,24) = 0.29; *p* = .749], total number of button presses [*F*(2,24) = 2.31; *p* = .120] and timing errors [*F*(2,24) = 0.80; *p* = .457]. However, with regard to the attention task a main effect of *Attention* was observed [*F*(2,24) = 5.22; *p* < .05]. Pairwise comparisons showed lower counting rates for the *AM* condition compared to the *AV* condition [*t*(12) = 4.22; *p* =.001]. However, the effect size of this effect is low (ŋ^2^ = 0.30). No differences were obtained comparing *AS* to *AM* [*t*(12) = −1.43; *p* =.176] or *AS* to *AV* [*t*(12) = 1.50; *p* =.158]*.* Taken together, no fundamental differences of task demands were observed between the three attention conditions.

**Table 1:** **Behavioral results for all three attention conditions** (***AS***, ***AM***, ***AV***)

	**Attention Sounds** (***AS***)	**Attention Motor** (***AM***)	**Attention Visual** (***AV***)
***Self****-****initiation task***			
Interval button presses (ms)	6233 (386)	6153 (425)	6188 (359)
Number of button presses	29.23 (2.8)	31.30 (2.5)	29.38 (2.78)
Timing errors (%)	2.33 (4.16)	2.94 (3.91)	1.06 (2.88)
***Attention task***			
Counting rates (%)	98.37 (4.59)	99.34 (3.71)	97.24 (3.59)

SD is given in parentheses.

### Electrophysiological data

**Figure 1:**
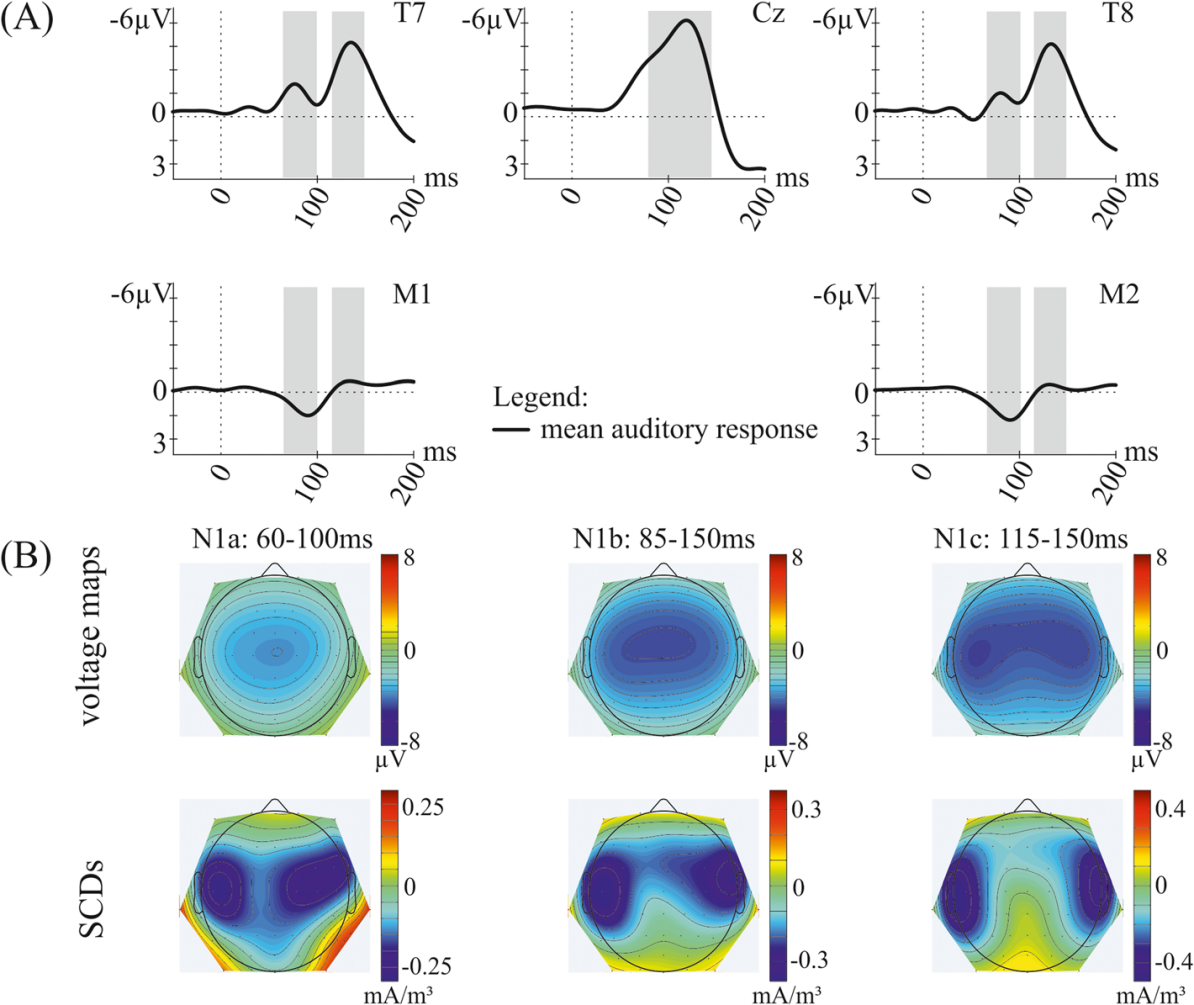
**Illustration of the mean auditory response.** (**A**) Grand-average ERPs (mean of attention conditions *AS*, *AM*, *AV* as well as self-initiated and externally-initiated sounds) at temporal and central electrodes and the mastoids. Analysed time windows are marked in grey. (**B**) Voltage maps and scalp current densities (SCDs) during the latency ranges of the N1a (60–100 ms), N1b (85–150 ms) and N1c (115–150 ms) time window. Note that only part of the baseline is included to the graphs.

In Figure 1A the grand-average auditory response across all conditions is depicted at central, temporal and mastoid electrodes. The ERP waveform shows a negative deflection in the typical N1 latency range at Cz and a double-peaked N1 at temporal electrodes with polarity inversion at the mastoids for only the early peak. Voltage maps and scalp current densities (Figure 1B) show the corresponding distributions for this deflection over the scalp in the N1b (85–150 ms), the N1a (60–100 ms) and the N1c (115–150 ms) time window, respectively. In the following, modulations of this auditory response caused by self-initiation and attention are reported. Statistical results for all time windows are presented in Table 2. Most importantly, for all three N1 time windows no interaction of the experimental factors *Production* and *Attention* was found (N1b window: *F*(2,24) = 0.85; *p* = .407; N1a window: *F*(2,24) = 6.24; *p* = .536; N1c window: *F*(2,24) = 0.80; *p* = .430). Thus, auditory N1 effects due to self-initiation and due to the allocation of attention for each time window will be presented separately (see Additional file 1 for grand-average ERPs as well as voltage maps and scalp current densities (SCDs) of single attention conditions *AS*, *AM*, *AV*). Furthermore, no interaction of *Attention* × *Production* was observed for the analysis of the mastoids [*F*(2,24) = 0.72; *p* = .495]. Thus, effects due to attention and self-initiation will be discussed separately as well.

**Table 2:** Results of the ANOVA for all N1 time windows

	**N1b time window** (**85**–**150 ms**)			**N1a time window** (**60**–**100 ms**)			**N1c time window** (**115**–**150 ms**)		
	***F***	***p***	**ŋ**^**2**^	***F***	***p***	**ŋ**^**2**^	***F***	***p***	**ŋ**^**2**^
Attention^2^	32.45	**	.730	10.57	**	.468	38.39	**	.762
Production^1^	18.31	**	.604	1.61	.228	.118	24.95	**	.675
Laterality^3^	38.46	**	.762	36.71	*	.754	10.37	**	.464
AnteriorPosterior^2^	6.32	*	.345	7.96	**	.339	3.80	.062	.241
Attention x Production^2^	0.85	.407	.066	6.24	.536	.049	0.80	.430	.063
Attention x Laterality^4^	9.65	**	.446	4.82	*	.287	9.93	**	.453
Attention x AnteriorPosterior^3^	17.83	**	.598	6.37	*	.347	9.02	**	.434
Production x Laterality^3^	30.85	**	.720	3.02	.076	.201	11.97	**	.499
Production x AnteriorPosterior^2^	4.05	.058	.253	2.90	.093	.194	3.76	.071	.239
Laterality x AnteriorPosterior^4^	2.03	.123	.145	4.71	*	.282	2.91	*	.196
Attention x Laterality x AnteriorPosterior^5^	1.69	.155	.123	1.70	.150	.124	2.53	*	.174
Production x Laterality x AnteriorPosterior^4^	2.85	*	.192	2.61	.051	.179	1.74	.168	.127
Attention x Production x Laterality^4^	1.92	.148	.138	1.12	.349	.085	1.56	.215	.115
Attention x Production x AnteriorPosterior^3^	0.66	.548	.052	0.53	.618	.042	0.43	.657	.034
Attention x Production x Laterality x AnteriorPosterior^5^	1.15	.340	.088	1.18	.324	.090	1.24	.295	.094

*F* values, *p* values and partial ŋ^2^ for each N1 time window are reported.

^1^*F*(1,12).

^2^*F*(2,24).

^3^*F*(4,48).

^4^*F*(8,96).

^5^*F*(16,192).

** *p* ≤ .001.

* *p* ≤ .05.

### Self-initiation effects on the auditory N1

**Figure 2:**
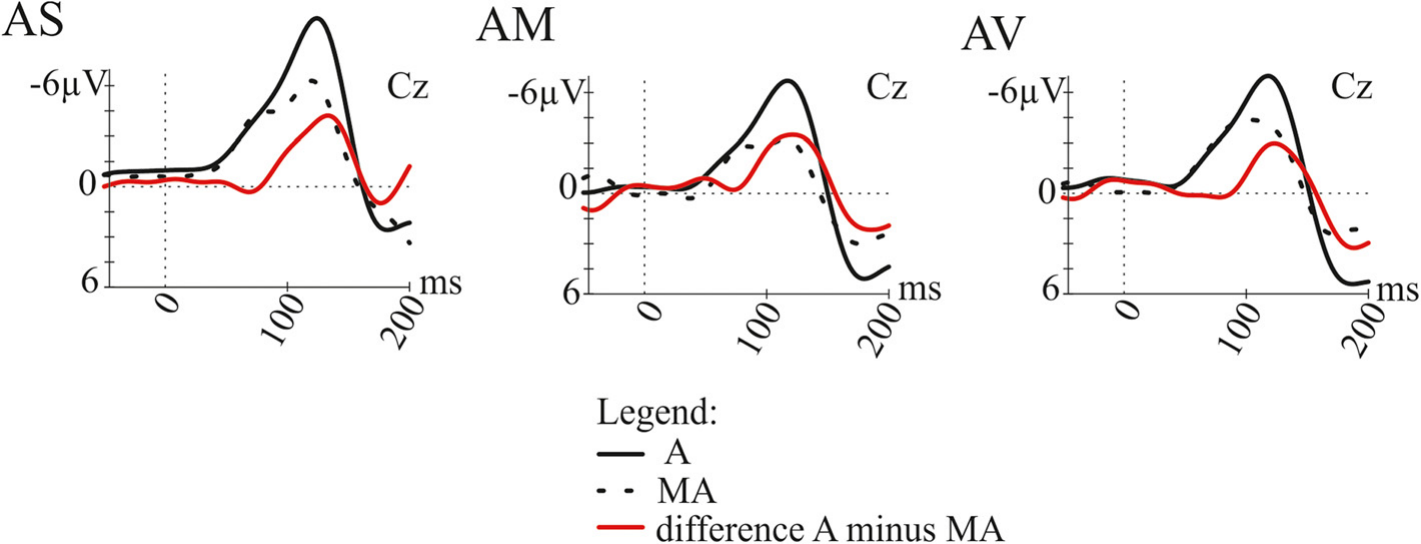
**Illustration of the self**-**initiation effect for single attention conditions.** Grand-average ERPs at Cz elicited by externally-initiated sounds (black solid line), self-initiated sounds (black dotted line) and the difference waves (externally-initiated minus self-initiated, red line), separately for the single attention conditions *Attention Sounds* (*AS), Attention Motor* (*AM)* and *Attention Visual (AV).*

**Figure 3:**
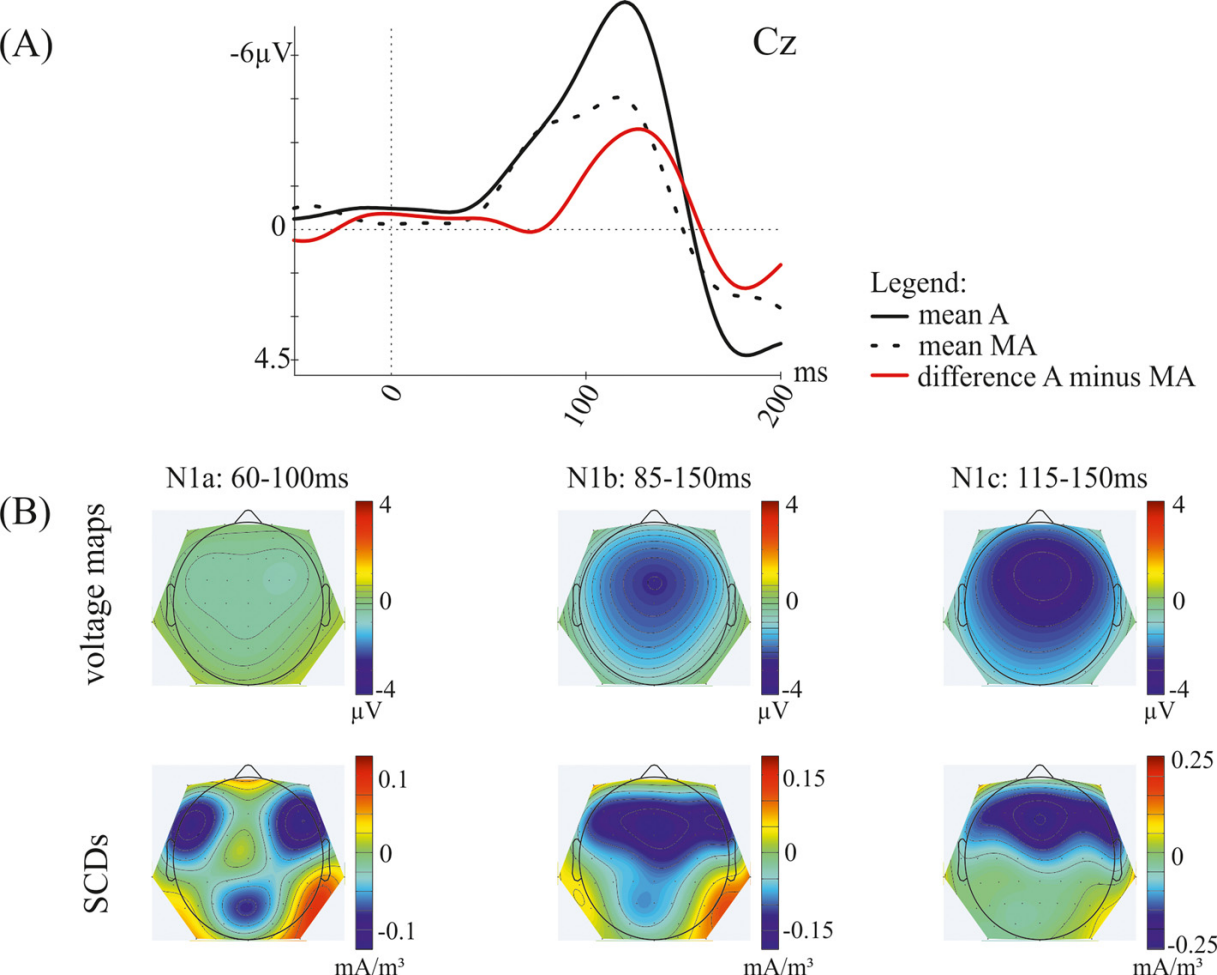
**Illustration of the mean self**-**initiation effect.** (**A**) Grand-average ERPs (mean of all attention conditions *AS*, *AM*, *AV*) at Cz elicited by externally-initiated sounds (black solid line), self-initiated sounds (black dotted line), as well as the difference wave (externally-initiated minus self-initiated, red line). (**B**) Voltage maps and scalp current densities (SCDs) of the difference wave during the latency ranges of the N1a (60–100 ms), N1b (85–150 ms) and N1c (115–150 ms) time window.

In Figure 2 grand-average ERP waveforms at Cz elicited by externally-initiated sounds and self-initiated sounds as well as the self-initiation effect (externally-initiated minus self-initiated) are shown, separately for the three attention conditions. Since comparable self-initiation effects were obtained in all attention conditions (*AS*, *AM*, *AV*) the mean of all three attention conditions was calculated and used for the further analysis. Figure 3A shows the grand-average ERP waveforms at Cz for the mean of all three attention conditions (*AS*, *AM*, *AV*) elicited by externally-initiated sounds and self-initiated sounds as well as the self-initiation effect (externally-initiated minus self-initiated). Furthermore, voltage maps and scalp current densities (SCDs) show the corresponding distribution over the scalp of the mean self-initiation effect in all three N1 time windows (Figure 3B). The analysis for the N1b time window revealed a main effect of *Production* [*F*(1,12) = 18.31; *p* = .001]. Also for the N1c time window a significant main effect [*F*(1,12) = 24.95; *p* < .001] was observed. This main effect of *Production* for both time windows was caused by lower amplitudes for self-initiated sounds compared to externally-initiated sounds. However, for the N1a time window no main effect of *Production* was found [*F*(1,12) = 1.61; *p* = .228], showing comparable amplitudes for self-initiated and externally-initiated sounds. Furthermore, for the N1b time window an interaction of *Production* × *Laterality* × *Anterior-Posterior* [*F*(8,96) = 2.85; *p* = .039] was obtained. Pairwise comparisons revealed lower amplitudes at frontal and central electrodes (*p* < .05 for F3, F4, Fz, F7, F8, C3, Cz, C4) for self-initiated compared to externally-initiated sounds, indicating a fronto-central distribution of the self-initiation effect (see Figure 3B, upper panel). The SCD topography of this effect also shows a pattern pointing at a fronto-central effect (see Figure 3B, lower panel). For the N1c time window no such interaction was observed [*F*(8,96) = 1.74; *p* = .168]. However, the analysis revealed an interaction of *Production* × *Laterality* [*F*(4,48) = 11.97; *p* = .001], showing a more central than lateral distribution of the self-initiation effect (see Figure 3B, upper panel). Again, the SCD distribution supports a fronto-central effect (see Figure 3B, lower panel). Contrary, for the N1a time window no interaction with the experimental factor *Production* was found. Additionally, at the mastoids no main effect of *Production* was obtained [*F*(1,12) = 2.98; *p* = .110].

### Comparison of self-initiation and attention effects on the auditory N1

**Figure 4:**
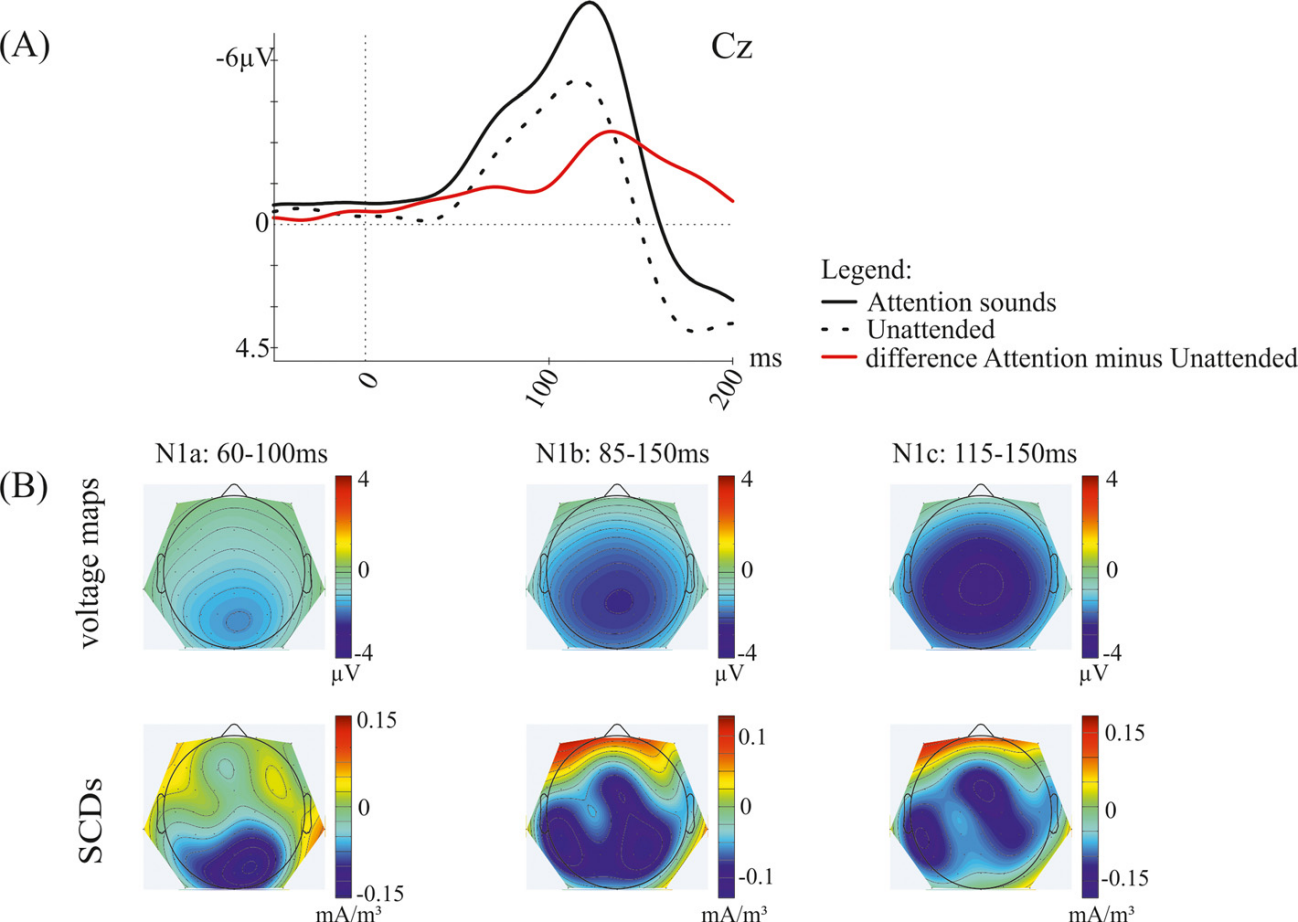
**Illustration of the attention sound effect.** (**A**) Grand-average ERPs (mean of self-initiated and externally-initiated sounds) at Cz elicited by attending the sounds (black solid line), not attending the sounds (black dotted line), as well as the difference wave (attended minus unattended, red line). (**B**) Voltage maps and scalp current densities (SCDs) of the difference wave during the latency ranges of the N1a (60–100 ms), N1b (85–150 ms) and N1c (115–150 ms) time window.

In the following, attention effects are outlined and then compared to the self-initiation effect. In order to simplify the comparison, we focused on effects of attending (AS) vs. not attending (AM, AV) to sounds, pooling the attention effects for the AM and AV conditions, which were rather similar (cf. Additional file 2). Thus, we compared effects of attending to sounds (AS vs. [AM+AV]/2 [over all production conditions]) to effects of self-initiating the sounds (A-MA [over all attention conditions]). Figure 4A shows the grand-average ERP waveforms at Cz elicited when attending the sounds and when not attending the sounds as well as the attention effect (attended minus unattended) for the mean of self-initiated and externally-initiated sounds. Furthermore, voltage maps and SCDs show the corresponding distribution over the scalp of the attention effect in all three N1 time windows (Figure 4B). The analysis for all N1 time windows revealed a main effect of *Attention* (N1b time window: *F*(2,24) = 32.45; *p* < .001; N1a time window: *F*(2,24) = 10.57; *p* = .001; N1c time window: *F*(2,24) = 38.39; *p* < .001). Pairwise comparison indicated higher activity for attending the sounds compared to not attending the sounds (N1b time window: *t(12)* = −7.87; *p* < .001; N1a time window: *t(12)* = −4.89; *p* < .001; N1c time window: *t(12)* = −8.28; *p* < .001). There was also a significant interaction of *Attention* × *Laterality* for the N1b time window [*F*(8,96) = 9.65; *p* < .001] and the N1a time window [*F*(8,96) = 4.82; *p* < .01]. Pairwise comparisons for the N1b time window showed higher amplitudes for attended compared to unattended sounds for all laterality levels [far left (*t(12)* = −6.29; *p* < .001), left (*t(12)* = −8.01; *p* < .001), midline (*t(12)* = −8.90; *p* < .001), right (*t(12)* = −7.50; *p* < .001), far right (*t(12)* = −4.72; *p* < .001)]. For the N1a time window the post-hoc analysis indicated higher amplitudes for attended compared to unattended sounds for all laterality levels except the far right (F8, T8, P8) level [far left (*t(12)* = −3.23; *p* < .05), left (*t(12)* = −5.03; *p* < .001), midline (*t(12)* = −6.16; *p* < .001), right (*t(12)* = −6.14; *p* < .001), far right (*t(12)* = −2.54; *p* = .130)]. For both time windows the attention effect shows a more parietal distribution (see Figure 4B, upper panel) compared to the self-initiation effect (see Figure 3B, upper panel).

The SCD distribution reveals that the putative sources of this attention effect are located in more parietal areas compared to the self-initiation effect (see Figure 3B, lower panel). However, the distribution for the N1b time window shows a more widespread activity than the distribution of the N1a time window (see Figure 4B, lower panel). Furthermore, there was a significant interaction of *Attention* and *Anterior-Posterior* for the N1b [*F*(4,48) = 17.83; *p* < .001] and the N1a [*F*(4,48) = 6.37; *p* < .01] time window. Pairwise comparisons revealed higher activity for attended compared to unattended sounds for all levels of both time windows [N1b time window: frontal (*t(12)* = −5.83; *p* < .001), central (*t(12)* = −7.86; *p* < .001), parietal (*t(12)* = −8.72; *p* < .001); N1a time window: frontal (*t(12)* = −2.91; *p* = .039), central (*t(12)* = −4.37; *p* < .01), parietal (*t(12)* = −5.91; *p* < .001)]. Again, this attention effect shows a parietal distribution (see Figure 4B, upper panel) which is supported by a parietal pattern of activity in the SCDs (see Figure 4B, lower panel). For the N1c time window no such interactions were found. However, the analysis revealed an interaction of *Attention* × *Laterality* × *Anterior-Posterior* [*F*(16,192) = 2.53; *p* < .05] for this time window, indicating a parietal and left-lateralized distribution of the attention effect which shows a more anterior distribution than the N1b and the N1a time window (see Figure 4B, upper panel). This finding is also supported by the SCDs which point at a more central topography (see Figure 4B, lower panel). Finally, at the mastoids no main effect of *Attention* was found [*F*(2,24) = 1.03; *p* = .374].

## Discussion

In the present study we investigated to which extent the N1-suppression effect for self-initiated sounds can be explained by a differential allocation of attention to self-initiated and externally-initiated sounds. To overcome possible limitations of the traditional blocked design self-initiated sounds and externally-initiated sounds as well as the motor control were presented within the same block. The allocation of attention was manipulated block-wise in three different attention conditions (*AS*, *AM*, *AV*), so that attention was directed to the sounds or was directed away from the sounds towards the own motor behavior or the visual stimulation. Moreover, we compared effects of self-initiation with attention effects to determine whether the underlying neural processes affect the same or different structures.

Horvath and colleagues (2012) have proposed that that N1 suppression might possibly be caused by split attentional resources in active conditions compared to passive conditions of the traditional blocked design [8-14]. We found an attenuation of the auditory N1 for self-initiated compared to externally-initiated sounds that was independent from the allocation of attention. That is, the N1 suppression was the same, irrespective of whether attention was directed to the sounds, directed to the motor act or directed to the visual stimuli. Thus, the N1-suppression effect cannot be explained by attentional differences between self- and externally-initiated sounds. In other words, sensory suppression to self-initiated sounds cannot be explained by the fact that the motor act draws away attention from auditory processing. Our finding is consistent with a recent study reporting reduced N1 amplitude during self-vocalization using a selective attention task to assess the N1 component independent of the attention effect [28].

Similar to forward modeling effects in other species [29,30], it has been argued that the N1-suppression effect is a very basic and automatic phenomenon [17]. Horvath and colleagues (2012) showed that the auditory input seems to be attenuated for a short period after the motor act, even if there is no contingency between button press and sound. It seems that the sensory processing during self-initiation of sounds is merely affected by the concurrent motor act [31]. Our finding that the neural processes underlying the N1 suppression are not modulated by attention strongly supports the view that they are rather automatic. In fact, the definition of an automatic (versus a controlled) process is that it does not interfere with attention [32,33].

As predicted, the allocation of attention to the sounds resulted in an increase of the auditory N1, as compared to the N1 elicited by the sounds when attention was directed to the button presses or to the visual stimuli. This finding is consistent with results from previous studies [19,25,34-36]. However, previous studies often obtained a more fronto-central distributed auditory attention effect [34,37,38], whereas we obtained a more parietal distribution. Nevertheless, top-down controlled attention has been reported to involve temporo-parietal and superior parietal areas [39], which is consistent with the distribution of our attention effect.

Moreover, the comparison of the self-initiation effect and the attention effect revealed that partly separate N1 components [25] are affected. Whereas all N1 components (i.e. N1a, N1b, N1c) were modulated by attention, only the late part of the N1 (i.e. N1b, N1c) was suppressed by self-initiation. Thus, we conclude that the predictive modeling underlying the N1-suppression effect is not “only” attention in time [20,27] but a mechanism that is separable from a mere attentional mechanism. In the present report, the frontocentral peak of the N1b did not coincide with the time of polarity reversal at the mastoids, which occurred slightly earlier. The N1b component is known to receive contributions from both the tangentially oriented, sensory-specific component and the unspecific component of the N1 [25]. Because the unspecific component occurs later in time, its contribution tends to delay the peak of the N1b on frontocentral leads [40]. Thus, the window of analysis chosen here around the peak of the N1b probably receives its largest contribution from the unspecific N1 component. There were no self-initiation effects at the mastoids on the polarity-inverted N1 deflection. This finding suggests that a large part of the N1-suppression effect may be due to the suppression of the unspecific N1 component rather than the attenuation of sensory responses in auditory cortex as stipulated from internal predictive models theory. Thus, it could be speculated that the N1-suppression effect as measured in most ERP studies may largely reflect the fact that self-initiated sounds are less arousing compared to externally-initiated sounds. However, the lack of N1 suppression on the mastoids and on fronto-central electrodes at the time of polarity reversal at the mastoids in the present experiment does not necessarily imply that sensory responses are not attenuated by self-initiation in auditory cortex at all. Indeed, previous MEG studies, which specifically measure the activity of tangentially oriented sources on auditory cortex, have found N1 suppression for self-initiated sounds [8,12,17].

## Conclusions

We could show that the N1 suppression was equally large and of equal distribution when subjects directed their attention towards the sound and when the directed their attention away from the sounds, towards the button presses or the visual stimuli. Thus, the self-initiation effect can hardly be explained by the differential amount of attention devoted to self- and externally-triggered sounds. Instead, the present results support the notion that N1 suppression for self-initiated sounds seems to reflect the activity of an internal predictive mechanism. Whereas the effects of voluntary attention affect all N1 components, the self-initiation effect seems to be confined to the N1b and N1c components. The present mixed design provides a useful tool to measure genuine self-initiation effects.

## Methods

### Participants

Fifteen healthy volunteers (7 male, 1 left-handed) participated in the experiment. Two male participants had to be excluded from the analysis due to low signal-to-noise ratio. Mean age of the remaining thirteen participants was 22.92 years (range: 19 to 29 years). All participants reported normal hearing and normal or corrected-to-normal vision. None were taking any medication affecting the central nervous system. All participants received either course credit or payment for their participation. The experiment was undertaken with the understanding and written consent of each subject. The experimental protocol conformed to the Declaration of Helsinki and the ethics guidelines of the German Association of Psychology (ethics board of the Deutsche Gesellschaft für Psychologie, DGPs: http://www.dgps.de/dgps/aufgaben/ethikrl2004.pdf) and did thus not require any additional ethics approval.

### Experimental conditions

**Figure 5:**
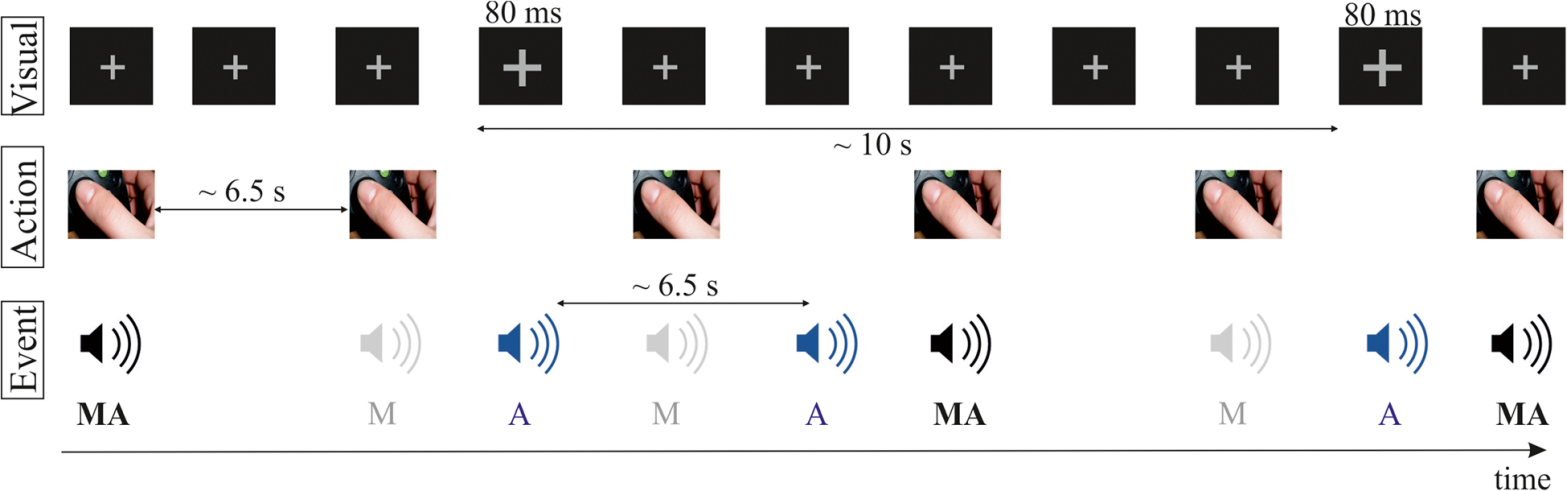
**Schematic illustration of the experimental mixed design.** Participants were asked to fixate on a grey cross constantly displayed on the center of a black screen. Small extensions of the fixation cross (from a visual angle of 0.69° to 0.74°) were presented for 80 ms duration. The extended fixation crosses were unpredictable in their occurrence using a variable SOA of 5–15 s. Additionally, participants pressed a button approximately every 6.5 s (range 5–8 s). With a probability of 50% button presses were followed by a sound immediately (MA, black). In the remaining 50% button presses were not followed by any sound (M, grey). It was not predictable if the button press would initiate a sound or not. Additionally, externally-produced sounds (with the same physical parameters as the self-initiated ones) occurred randomly between button presses (A, blue). Externally-produced sounds were unpredictable in their occurrence with a variable SOA between 5–8 s (mean of 6.5 s).

Participants were asked to fixate on a grey cross constantly displayed on the center of a black screen. Small extensions of the fixation cross (from a visual angle of 0.69° to 0.74° with a distance to the monitor of 100 cm) were presented for 80 ms duration using a variable stimulus onset asynchrony (SOA) of 5–15 s. These extended fixation crosses were not predictable for the participants. Using a mixed experimental design self-initiated and externally-initiated sounds were presented in the same block (Figure 5). Participants were instructed to press a button with their left or right thumb (depending on handedness) with self-paced intervals of 5–8 s (mean: 6.5 s). In 50% of the trials button presses initiated a 50 ms sine tone of 1000 Hz (including 10-ms rise and 10-ms fall times) which was presented immediately after the button press through headphones (Sennheiser HD 25–1) (motor-auditory condition in the blocked design, MA). The intensity of the sounds was adjusted to a comfortable loudness by the participant with soft foam earplugs inserted to attenuate any other sounds. In the remaining 50% of the trials button presses were not followed by any sound (motor-only condition in the blocked design, M). For the participants it was not predictable whether the button press would initiate a sound or not. Additionally, externally-initiated sounds (with the same physical parameters as the self-initiated sounds) were presented randomly between button presses (auditory-only condition in the blocked design, A). Externally-initiated sounds were unpredictable in their occurrence. The SOA between two externally-initiated sounds ranged randomly between 5–8 s. All sounds were generated with MATLAB (http://www.mathworks.com). To avoid a possible overlap with preceding self-initiated sounds, externally-initiated sounds were always presented at least 1 s after the occurrence of a button press. When the SOA between a preceding externally-initiated sound and a button press (initiating a sound or not) was smaller than 1 s both trials were excluded, but the respective number of trials were added at the end of the block to avoid loss of data. In addition to the self-initiation task the allocation of attention was manipulated block-wise. Three attention conditions were included (*Attention Sound*, *Attention Motor*, *Attention Visual*). In the *Attention Sound (AS)* condition participants were instructed to count all sounds they could hear, including self-initiated and externally-initiated ones. In the *Attention Motor (AM)* condition participants counted all button presses they made. In the *Attention Visual (AV)* condition they were asked to count all extended fixation crosses they saw on the screen. Thus, less attention should be directed to the sounds when participants attend to the motor act or to the visual stimuli than when they attend to the sounds.

### Experimental procedure

During EEG recordings, participants were seated in a sound-attenuated and electrically shielded chamber. Auditory stimulation was run via MATLAB using the Cogent2000 toolbox (http://www.vislab.ucl.ac.uk/cogent_2000.php). Participants were instructed to press the button once every 5–8 s (mean: 6.5 s). They were informed that a button press would be followed by a sound or silence. Participants were informed about the occurrence of the externally-initiated sounds. However, they were not provided with further information about them. To get used to the self-initiation task participants received several training blocks before the experiment. In these training blocks visual feedback of the button press SOA was given after each button press. In the main experiment visual feedback about the mean button press interval and the responses that were too slow or too fast were only shown at the end of each block. To avoid data loss, a block was repeated whenever participants pressed the button more than 5 times too slow or too fast within one block. In addition to the self-initiation task, participants had to count either all the sounds they could hear (*AS*), all the button presses they made (*AM*) or all the extended fixation crosses they saw (*AV*). Participants were always informed before the beginning of each block about the respective task. After each block they reported the number of counted events. To make sure participants attended to the particular events effectively the block was repeated whenever they miscounted more than +/− 2. Meta-blocks, including all three attention conditions, were repeated eight times. Thus, the EEG experiment consisted of twenty-four experimental blocks. In the meta-blocks the attention conditions (*AS*, *AM*, *AV*) were pseudo-randomized.

Each block consisted on average of twelve (range: ten to fourteen) self-initiated sounds (MA) and silent button presses (M), respectively. This variation was included to make the counting task less predictable for the participants. A comparable number of externally-initiated sounds (A) was presented depending on the mean SOA of the self-paced button presses. In total a mean of 96 trials were analysed for each event (MA, A, M) for each attention condition (*AS*, *AM*, *AV*), respectively.

### Data recording and analysis

EEG activity was recorded continuously with Ag/AgCl electrodes from 60 standard locations (Fp1, Fp2, AF3, AFz, AF4, F7, F5, F3, F1, Fz, F2, F4, F8, FT7, FC3, FC1, FCz, FC2, FC4, FC6, FT8, T7, C5, C3, C1, Cz, C2, C4, C6, T8, TP7, CP5, CP3, CP1, CPz, CP2, CP4, CP6, TP8, P7, P5, P3, P1, Pz, P2, P4, P6, P8, PO9, PO7, PO3, POz, PO4, PO8, PO10, O1, Oz, O2) according to the international 10–20 electrode system [41] including the left and right mastoid (M1, M2). An additional electrode was placed at the tip of the nose (serving as offline reference). EOG was measured using the setup described by [42] with one electrode at nasion and two electrodes at the outer canthi. EEG signals were sampled at 500 Hz.

Automatic eye movement correction was applied on the data according to the procedure described in [42], preceded by a 1 to 100 Hz offline band-pass filter. After EOG artifact correction, data were filtered with a 1–25 Hz band-pass filter (kaiser-window, ripple: 0.017, length: 5653 points). For each trial, an epoch of 600 ms duration including a 200 ms pre-stimulus baseline was extracted from the continuous EEG record. Epochs with amplitude changes exceeding 75 μV on any channel were rejected from further analysis. ERPs were averaged time-locked to stimulus onset separately for each event type, attention condition and participant. Button press errors (inter-press interval < 5000 ms or > 8000 ms) were removed from the EEG analysis.

To correct for motor activity present in responses to self-initiated sounds, the ERPs elicited by button presses followed by no sound were subtracted from the ERPs elicited to the self-initiated sounds. This motor-response-corrected ERP was then compared with the ERP of the externally-initiated sounds. In all figures and analysis, ERPs elicited by the self-initiated sounds were corrected this way. This approach has become an appropriate procedure in previous research (presenting MA and M conditions in separate blocks) to measure auditory processing activity in the presence of motor-related activity. However, presenting MA and M conditions introduces a possible confound, namely that it cannot be completely ruled out that non-motor responses, e.g. responses related to temporal expectations of the sound, might also be eliminated subtracting the ERPs elicited by button presses followed by no sound from the ERPs elicited to the self-initiated sounds. However, as the N1-suppression effect observed in the present study was virtually identical to the one reported in previous studies using no mixed design suggests that the suppression effects are not an artefact of the subtraction method of the mixed design.

Because of the multiple components with separate and potentially overlapping latencies underlying the N1 [25] we investigated three separate intervals in the N1 latency range which fit to the peaks N1a, N1b and N1c that have been described in the literature before [25,26,40,43]. Intervals for the N1a and N1c peaks were defined to encompass the first and second peak of the N1 at temporal electrodes. The interval for the N1b peak was defined to encompass the broader N1 peak at central and frontal electrodes. Thus, ERP effects were investigated around the grand-average peaks in the latency range of 85–150 ms (N1b time window), 60–100 ms (N1a time window) and 115–150 ms (N1c time window) after stimulus onset (see Figure 1). ERP amplitudes were calculated from the individual averages as the mean amplitude within these specified analysis time windows. A repeated measurement analysis of variance (ANOVA) with the factors *Attention* (AS, AM, AV), *Production* (self-initiated vs. externally-initiated), *Laterality* (far left: F7, T7, P7; left: F3, C3, P3; midline: Fz, Cz, Pz; right: F4, C4, P4; far right: F8, T8, P8) and *Anterior-Posterior* (frontal: F7, F3, Fz, F4, F8; central: T7, C3, Cz, C4, T8; parietal: P7, P3, Pz, P4, P8) was computed for each N1 time window, on the mean amplitudes of the electrodes F7, T7, P7, F3, C3, P3, Fz, Cz, Pz, F4, C4, P4, F8, T8, P8. Moreover, in order to identify the sensory specific N1 component generated in auditory cortex, a further repeated measurement ANOVA with the factors *Attention* × *Production* was calculated for the mastoid signals in the latency range of 70-110 ms, since the generator for this component has a tangential orientation and results in N1 responses which are negative over frontocentral locations but are also recorded with inverted polarity on the mastoids.

For studying the scalp topographies in the interesting latency ranges, ERP voltage distributions were transformed into scalp current density (SCD) distributions, computing the second spatial derivative of the interpolated potential distribution [44,45]. The maximum degree of the Legendre polynomials was chosen to be 50, and the order of splines (m) was set to 4. A smoothing parameter lambda of 10^−4^ was applied. For behavioural data a one-way repeated ANOVA with the factor *Attention* was computed to compare inter-press time intervals, total number of button presses and timing errors for the self-initiation task between the attention conditions (*AS*, *AM*, *AV*). Furthermore, the counting rates of the attention task for all attention conditions were compared. The counting rates represent the total number of correctly counted events in relation to the total number of actual events of each attention condition. Greenhouse-Geisser correction was applied where appropriate. Additional pairwise comparisons (p-value alpha-adjusted using the Bonferroni correction) were conducted when appropriate to clarify the origin of significant effects. Only interactions that are relevant for the addressed question are reported.

## Endnotes

^a^As the recording of neural responses to motor activity without sounds in separate experimental blocks and subtracting these responses from the motor responses of the active condition could lead to biased estimates of sensory processing [15,17], we used a variant of the mixed N1 suppression paradigm, in which 50% of the button presses trigger a sound while the other 50% do not. With this, the representation of the motor command (efference copy) should be fully eliminated.

## Authors’ contributions

JT, IS, KS, and ES designed the study. JT, IS and KS programmed the task. JT acquired the data. JT, IS and KS performed the data analysis. All authors participated the data evaluation and interpretation and in writing the manuscript, and have approved the final version of the manuscript.

## Supplementary Material

Additional file 1 **Grand-average ERPs of single attention conditions.** Grand-average ERP waves elicited by externally-initiated sounds (black solid line) and self-initiated sounds (black dotted line), separately for the single attention conditions *Attention Sounds* (*AS), Attention Motor* (*AM)* and *Attention Visual (AV)* at temporal and central electrodes and the mastoids. The corresponding difference waves (externally-initiated minus self-initiated) are depicted in red. Voltage maps and scalp current densities (SCDs) of the difference wave during the latency ranges of the N1a (60–100 ms), N1b (85–150 ms) and N1c (115–150 ms) time window are also depicted. Click here for file

Additional file 2 **Attention effect for single attention conditions.** Voltage maps and scalp current densities (SCDs) of the attention effects for the single attention conditions *Attention Sounds* (*AS), Attention Motor* (*AM)* and *Attention Visual (AV)* during the latency ranges of the N1a (60–100 ms), N1b (85–150 ms) and N1c (115–150 ms) time window are depicted. Click here for file
