# Supplementary figures and images for: The N1-suppression effect for self-initiated sounds is independent of attention
Source: BMC Neurosci. 2013 Jan 3;14:2. doi: 10.1186/1471-2202-14-2 (PMC3573961; doi:10.1186/1471-2202-14-2)

Additional file 1:

AS

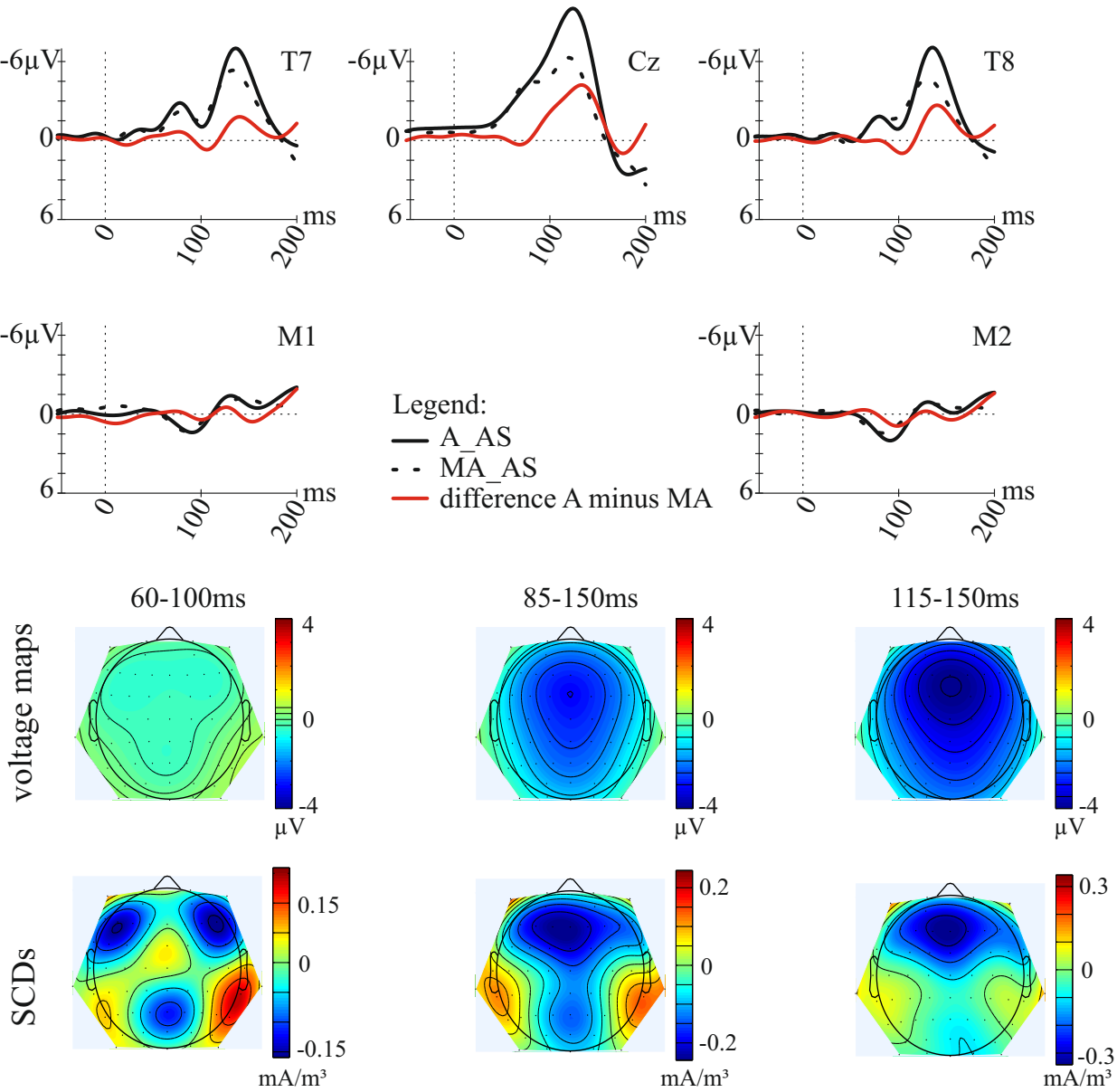

# AM

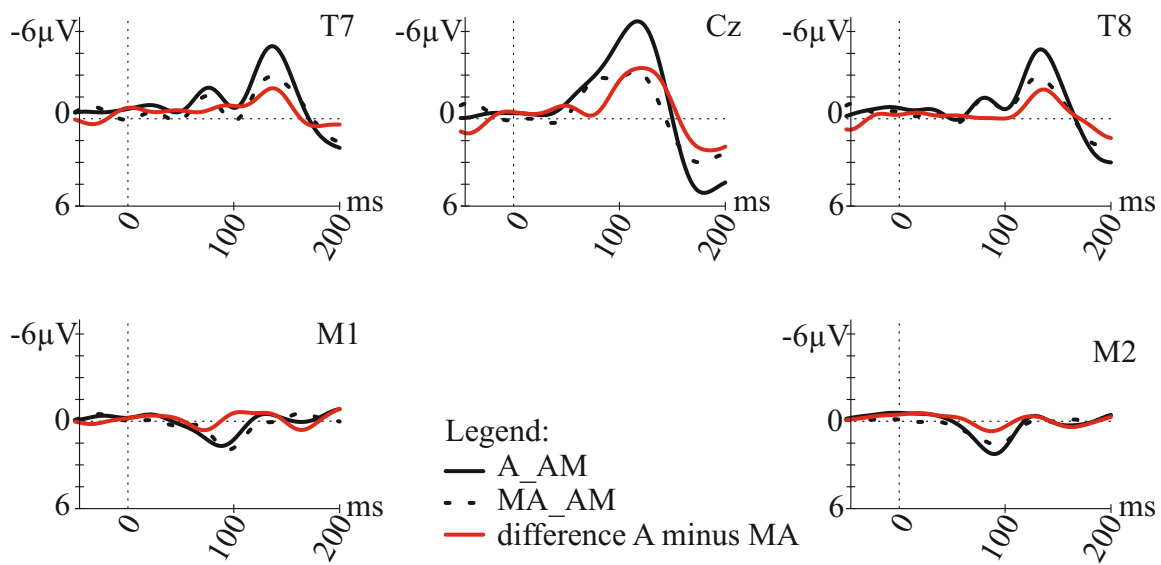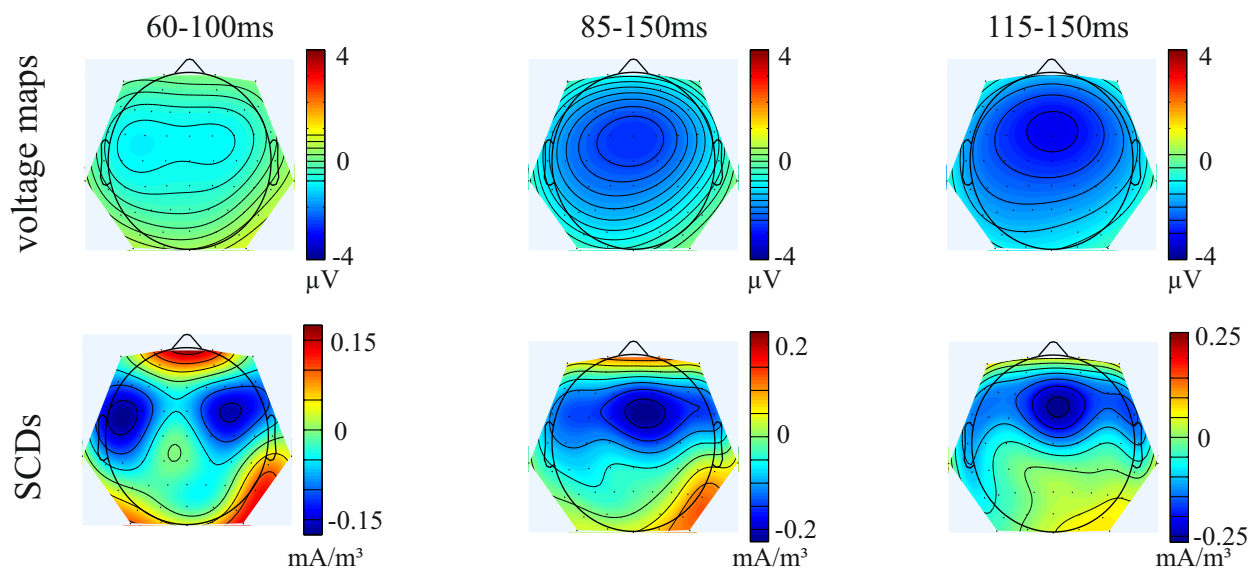

AV

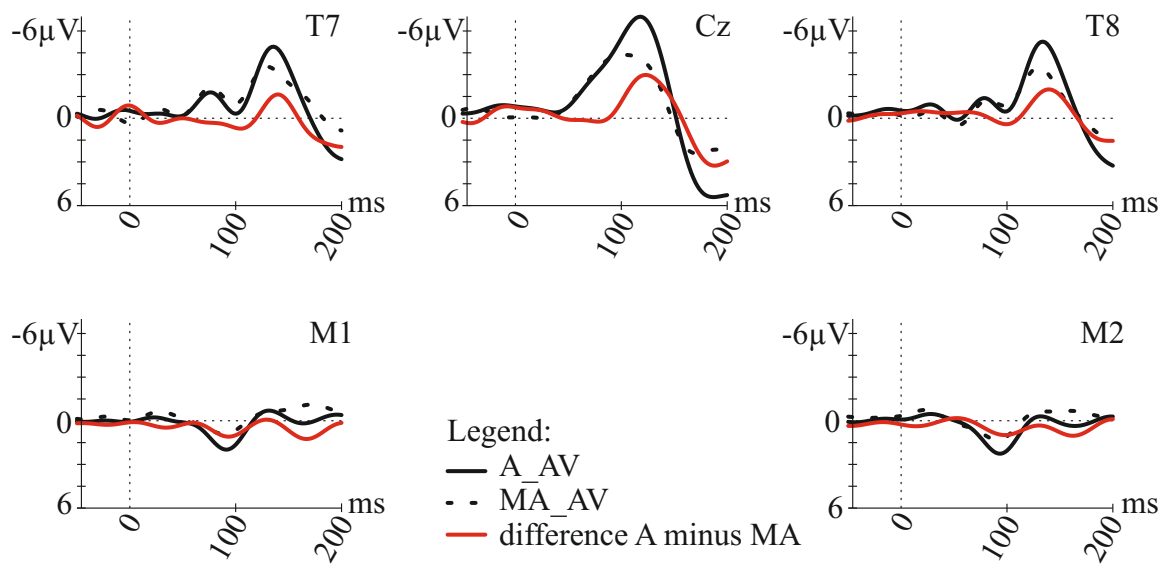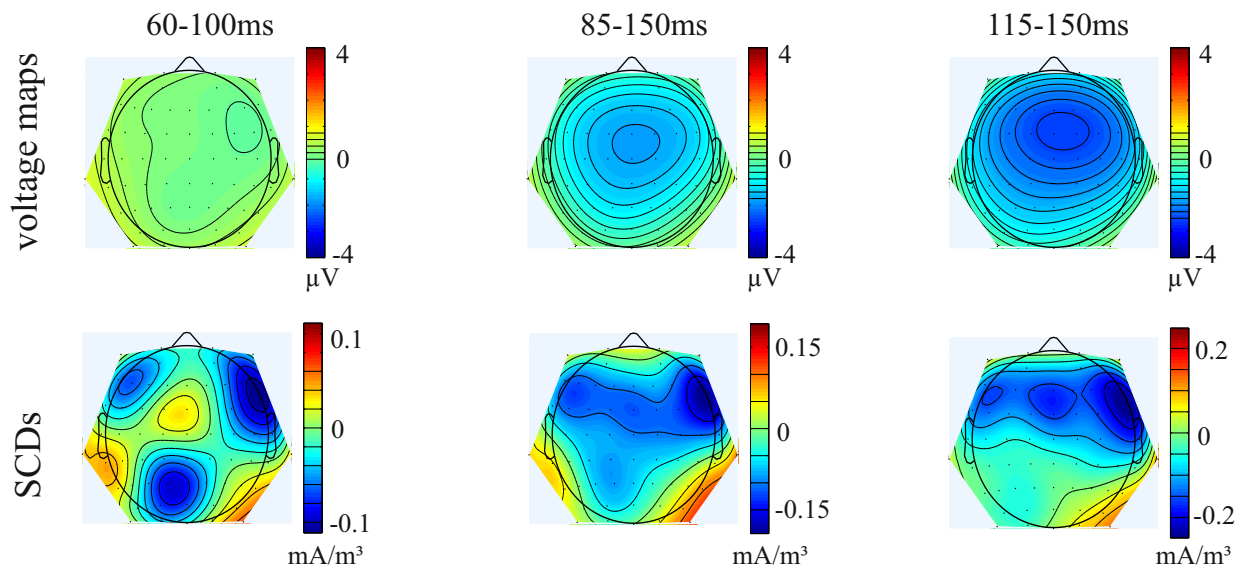

Supplement: Additional file 1 — Grand-average ERPs of single attention conditions. Grand-average ERP waves elicited by externally-initiated sounds (black solid line) and self-initiated sounds (black dotted line), separately for the single attention conditions Attention Sounds (AS), Attention Motor (AM) and Attention Visual (AV) at temporal and central electrodes and the mastoids. The corresponding difference waves (externally-initiated minus self-initiated) are depicted in red. Voltage maps and scalp current densities (SCDs) of the difference wave during the latency ranges of the N1a (60–100 ms), N1b (85–150 ms) and N1c (115–150 ms) time window are also depicted. [file 1471-2202-14-2-S1.pdf]

Additional file 2:

AS

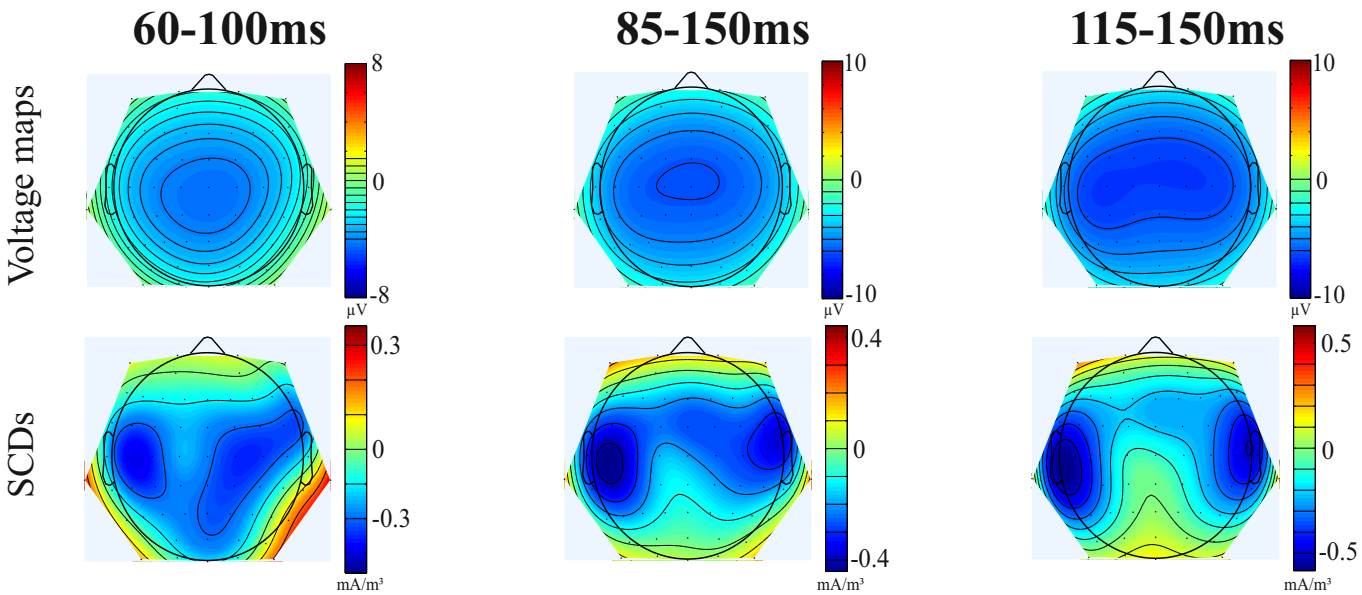

AM

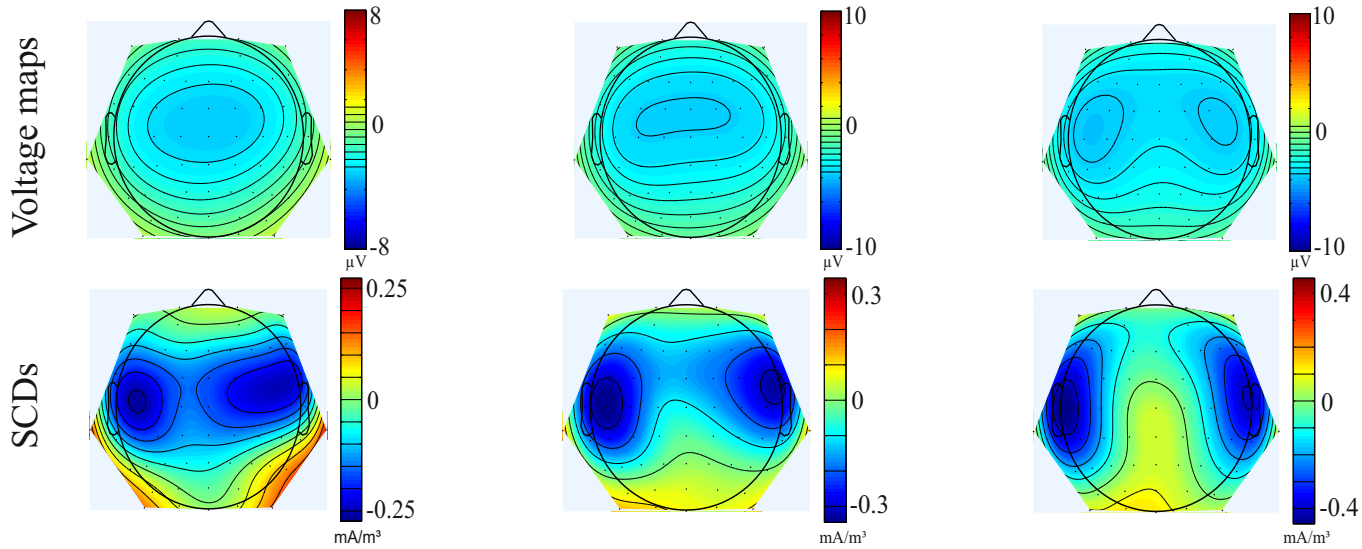

AV

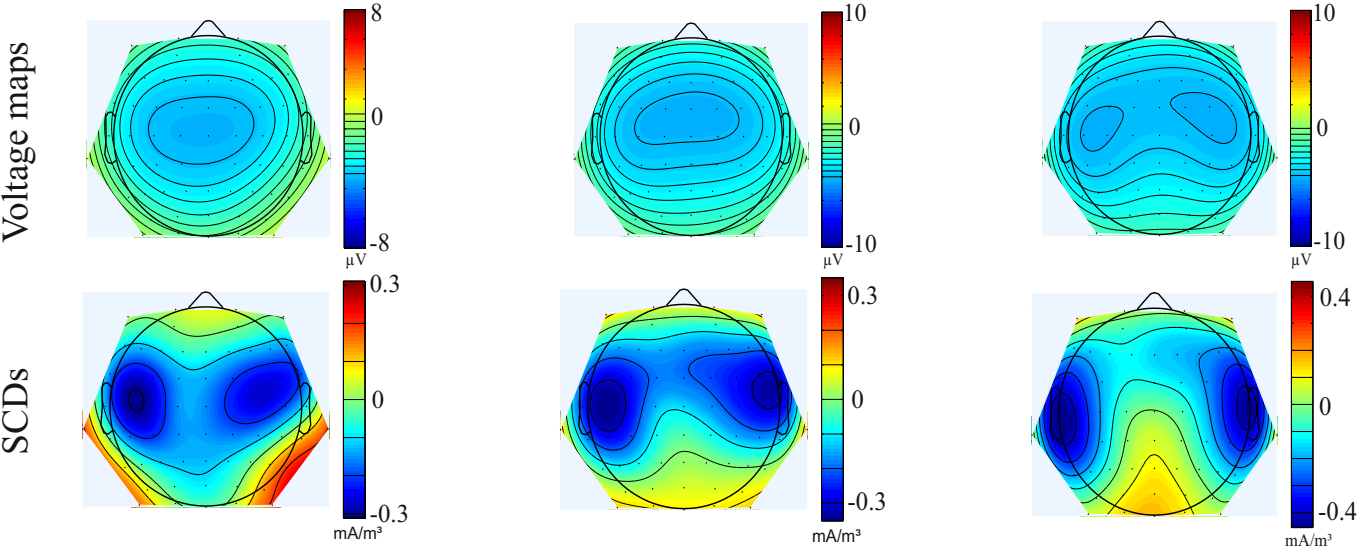

Supplement: Additional file 2 — Attention effect for single attention conditions. Voltage maps and scalp current densities (SCDs) of the attention effects for the single attention conditions Attention Sounds (AS), Attention Motor (AM) and Attention Visual (AV) during the latency ranges of the N1a (60–100 ms), N1b (85–150 ms) and N1c (115–150 ms) time window are depicted. [file 1471-2202-14-2-S2.pdf]
